# MiR‐34b‐3p represses cell proliferation, cell cycle progression and cell apoptosis in non‐small‐cell lung cancer (NSCLC) by targeting CDK4

**DOI:** 10.1111/jcmm.14404

**Published:** 2019-06-14

**Authors:** Hongxiang Feng, Feixiang Ge, Lanfang Du, Zhenrong Zhang, Deruo Liu

**Affiliations:** ^1^ Department of Thoracic Surgery China–Japan Friendship Hospital Beijing China; ^2^ Department of Cell Biology and Genetics, College of Life Sciences Nankai University Tianjin China; ^3^ Department of Emergency Peking University Third Hospital Beijing China

**Keywords:** apoptosis, CDK4, cell cycle, cell proliferation, miR‐34b‐3p, miRNA high throughput, NSCLC

## Abstract

Lung cancer is the most common incident cancer, with a high mortality worldwide, and non‐small‐cell lung cancer (NSCLC) accounts for approximately 85% of cases. Numerous studies have shown that the aberrant expression of microRNAs (miRNAs) is associated with the development and progression of cancers. However, the clinical significance and biological roles of most miRNAs in NSCLC remain elusive. In this study, we identified a novel miRNA, miR‐34b‐3p, that suppressed NSCLC cell growth and investigated the underlying mechanism. miR‐34b‐3p was down‐regulated in both NSCLC tumour tissues and lung cancer cell lines (H1299 and A549). The overexpression of miR‐34b‐3p suppressed lung cancer cell (H1299 and A549) growth, including proliferation inhibition, cell cycle arrest and increased apoptosis. Furthermore, luciferase reporter assays confirmed that miR‐34b‐3p could bind to the cyclin‐dependent kinase 4 (CDK4) mRNA 3′‐untranslated region (3′‐UTR) to suppress the expression of CDK4 in NSCLC cells. H1299 and A549 cell proliferation inhibition is mediated by cell cycle arrest and apoptosis with CDK4 interference. Moreover, CDK4 overexpression effectively reversed miR‐34‐3p‐repressed NSCLC cell growth. In conclusion, our findings reveal that miR‐34b‐3p might function as a tumour suppressor in NSCLC by targeting CDK4 and that miR‐34b‐3p may, therefore, serve as a biomarker for the diagnosis and treatment of NSCLC.

## 1 INTRODUCTION

Lung cancer is one of the most common aggressive cancers characterized by a high incidence and mortality worldwide. Non‐small‐cell lung cancer (NSCLC) is the major subtype of lung cancer, accounting for approximately 80% of lung cancer cases. Although treatment for NSCLC patients has made great progress in recent decades, the prognosis remains poor. Thus, there is a great demand to investigate the molecular mechanisms underlying NSCLC cell growth, metastasis and chemosensitivity to develop new therapies and treatment targets for NSCLC.

MicroRNAs (miRNAs) are endogenous non‐coding RNAs approximately 20‐24 nucleotides in length that regulate gene expression by binding to the 3′‐untranslated regions (3′‐UTRs) of target mRNAs. MicroRNAs have been reported to be involved in a series of biological processes, including cell cycle regulation, differentiation, metabolism, proliferation, apoptosis, invasion and angiogenesis.^1^, ^2^, ^3^ The dysregulation of certain miRNAs may lead to tumourigenesis in multiple cancer types,^4^ indicating that miRNAs may serve as key mediators in cancer development. Therefore, it makes sense to explore the functions and potential applications of miRNAs in cancer biology.

The aberrant expression of miRNAs regulates tumourigenesis, angiogenesis and tumour metastasis.^1^, ^4^ For example, down‐regulation of the let‐7 miRNA family was associated with a poor prognosis in patients with NSCLC.^5^ miRNA‐16 inhibits NSCLC cell growth and motility by targeting hepatoma‐derived growth factor (HDGF).^6^ miRNA‐146a suppresses cell growth by targeting EGFR.^7^ However, whether miR‐34b‐3p is involved in NSCLC tumour growth and its functional role remain elusive.

In this study, we investigated the underlying mechanism of miR‐34b‐3p in NSCLC. We found that CDK4 is a direct functional target of miR‐34b‐3p in NSCLC cell (A549 and H1299) growth and that miR‐34b‐3p suppresses NSCLC cell (A549 and H1299) growth by inhibiting CDK4 expression.

## 2 MATERIALS AND METHODS

### 2.1 Clinical data

Microarray data of the relative expression levels of miRNAs, including miR‐34b‐3p, in 100 NSCLC patients paired with adjacent normal tissues were obtained from the National Center for Biotechnology Information Gene Expression Omnibus (GEO) database (No. GSE64591). Human NSCLC cancer tissue samples were obtained from the Department of Thoracic Surgery, China‐Japan Friendship Hospital (Beijing, China) under ethical assessment.

### 2.2 Cell culture

Human lung cancer cell lines (A549 and H1299) and normal lung cells (BEAS‐2B) were obtained from the Institute of Biochemistry and Cell Biology of the Chinese Academy of Sciences (Shanghai, China). All cells were cultured in Dulbecco's modified Eagle's medium (DMEM, Sigma) supplemented with 10% foetal bovine serum (FBS, Sigma), 100 U/ml penicillin and 100 μg/ml streptomycin (pen/strep, Sigma). The cells were cultured at 37°C in the presence of 5% CO_2_.

### 2.3 Cell transfection

MiR‐34b‐3p mimics and a scrambled negative control (NC) were provided by Ribobio (Guangzhou, China). The sequences are as follows: MiR‐34b‐3p mimic sense (5′‐3′), CAAUCACUAACUCCACUGCCAU; and miR‐34b‐3p mimic antisense (5′‐3′), GGCAGUGGAGUUAGUGAUUGUU. A small interfering RNA (siRNA) against CDK4 (HSH054656) and a CDK4 plasmid (T7702) fused with an HA tag were constructed by FulenGen (Guangzhou, China). Cells were transfected with Lipofectamine 3000 reagent (Invitrogen, USA) according to the manufacturer's protocols.

### 2.4 Reverse transcription and quantitative real‐time PCR (qRT‐PCR)

Total RNA was extracted from tissues or cultured cells with TRIzol reagent according to the manufacturer's protocol (Life Technologies, UK). Quantitative real‐time polymerase chain reaction (qRT‐PCR) was performed on an ABI 7300 system (Applied Biosystems, USA) according to the manufacturer's instructions. The mRNA levels were detected by using SYBR Green PCR Master Mix, and miR‐34b‐3p levels were detected by using a TaqMan MicroRNA Assay Kit (P/N: 002102, Applied Biosystems). The relative expression of CDK4 and miR‐34b‐3p was normalized to that of the internal control (GAPDH and U6 respectively). Relative expression was calculated using the 2^‐△△Ct^ method. The sequences of the PCR primers were as follows: CDK4 forward AGTTCGTGAGGTGGCTTTA and reverse GGGTGCCTTGTCCAGATA.

### 2.5 Cell proliferation assay

Cell proliferation was examined with a Cell Counting Kit‐8 (CCK‐8, Dojindo, Japan) assay according to the manufacturer's protocols. A549 and H1299 cells were transfected with the corresponding plasmids or miRNAs and then seeded into 96‐well plates and cultured for 24, 48, 72 and 96 hours. Next, CCK‐8 reagent (approximately 10 μL) was added to each well and then incubated for an additional 1 or 2 hours. The absorbance at OD 450 was measured with a microplate reader. This experiment was performed in triplicate.

### 2.6 Cell cycle and apoptosis analysis

Cells were collected and fixed in 70% ethanol at 4°C overnight. Then, the cells were treated with RNase A (50 mg/mL) and stained with propidium iodide (PI, 25 mg/mL) for 30 minutes at 37°C after washing with PBS. The distribution of cell cycle phases was counted by a FACS Calibur Flow Cytometer (Becton, Dickinson and Company, CA). The phase ratio (%) was calculated as the percentage of cells in G1/S/G2 phase. Cell apoptosis was determined with an Annexin V‐FITC Apoptosis Detection Kit (BD Pharmingen, San Diego, CA). Cells were collected and resuspended in 200 μL of binding buffer. Following incubation with Annexin V‐FITC and PI in the dark at room temperature for 15 minutes, the apoptosis rate was detected using a FACS Calibur Flow Cytometer (Becton, Dickinson and Company). Each experiment was conducted in triplicate.

### 2.7 Luciferase reporter assay

A549 and H1299 cells were seeded and cultured on 24‐well plates. Then, cells were cotransfected with the miR‐34b‐3p mimic or miR‐NC together with the wild‐type (WT) or mutated (MUT) 3′‐UTR of CDK4. After 48 hours, cells were collected, and luciferase was detected by using a Dual Luciferase Reporter Assay (Promega Corporation, Madison, WI) according to the manufacturer's protocol. The firefly luciferase signal was normalized to that of the Renilla luciferase signal.

### 2.8 Western blot analysis

Total protein extracts from tissues and cultured cells were treated with RIPA buffer containing 3% proteinase inhibitor cocktail (Sigma) according to the manufacturer's instructions. Cell lysates were separated by electrophoresis on 10% sodium dodecyl sulfate polyacrylamide and transferred to polyvinylidene difluoride (PVDF) membranes (Invitrogen). The primary antibody against CDK4 (12790) was purchased from Cell Signaling Technology, Inc (Danvers, MA). Signals were detected on an enhanced chemiluminescence system (Amersham Biosciences, USA).

### 2.9 Statistical analysis

All data are shown as the mean ± standard deviation (SD). Student's *t* test was used to estimate significant differences between groups. *P* < 0.05 indicated a statistically significant difference.

## 3 RESULTS

### 3.1 MicroRNA‐34b‐3p expression is down‐regulated in NSCLC tissues and cell lines

**Figure 1:**
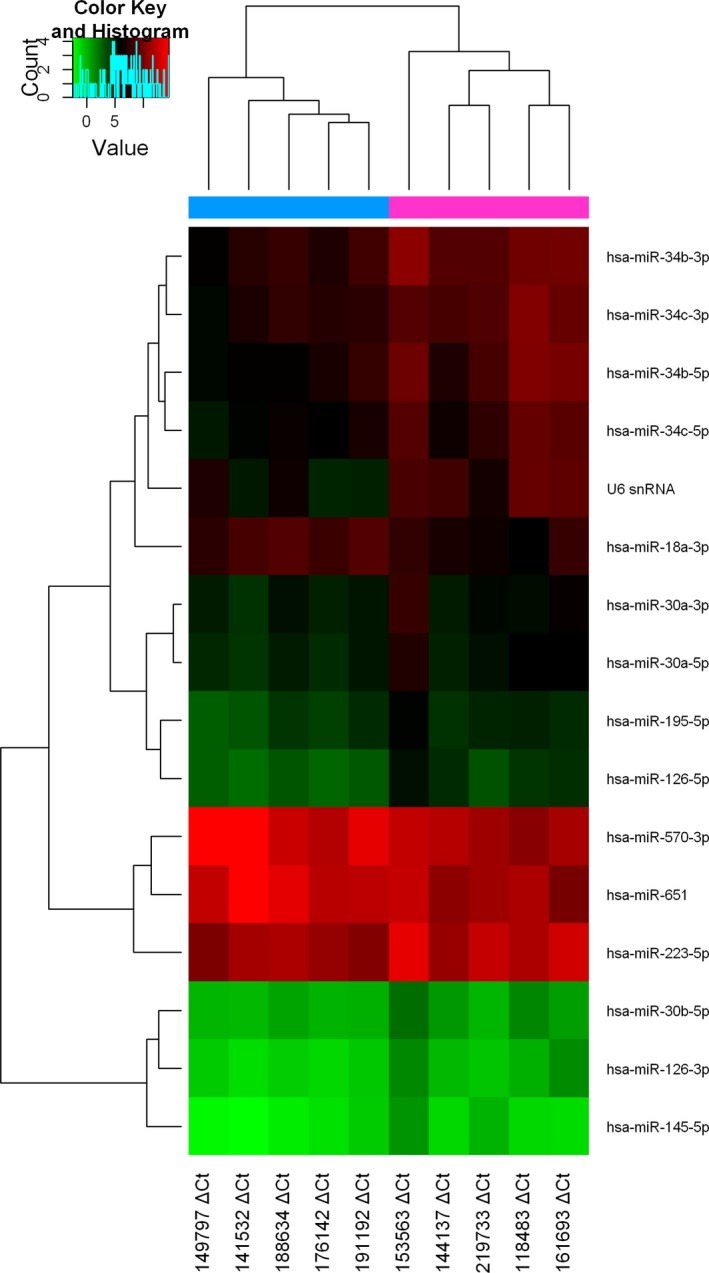
Hierarchical clustering of miRNAs in non‐small‐cell lung cancer (NSCLC) samples from the Gene Expression Omnibus database. The heatmap shows the results of the two‐way hierarchical clustering of miRNAs from NSCLC samples. The colour scale shown at the top illustrates the relative expression level of a miRNA across all samples. Deep red colour represents an expression level above the mean. Green colour represents an expression below the mean. Sample △Ct represents samples with a good prognosis vs those with a poor prognosis

**Figure 2:**
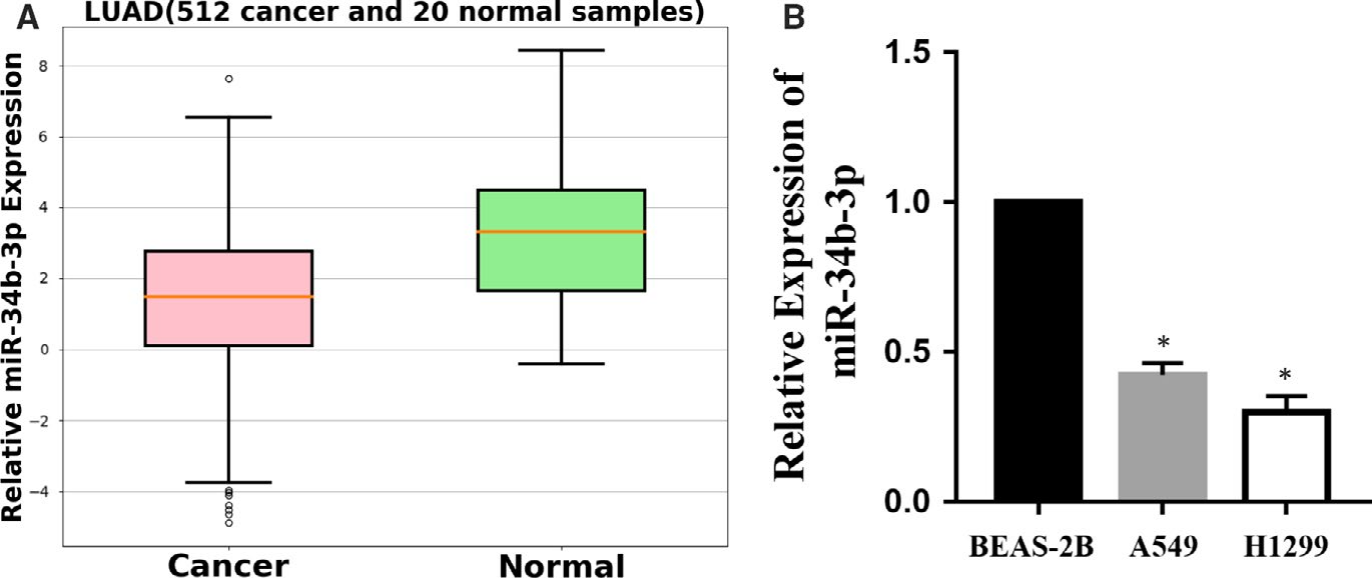
Expression of miR‐34b‐3p in non‐small‐cell lung cancer (NSCLC) tissues and cell lines. (A) qRT‐PCR examination of miR‐34b‐3p levels in lung adenocarcinoma tissues and adjacent normal tissues. (B) qRT‐PCR analysis revealed the expression of miR‐34b‐3p in two NSCLC cell lines (H1299 and A549) and in normal lung cells (BEAS‐2B). All experiments were performed in triplicate. *P < 0.05

To explore whether miR‐34b‐3p was detectable and altered in NSCLC, the expression patterns of miR‐34b‐3p were examined in paired tumour tissues and matched adjacent normal tissue of NSCLC from 100 patients at stages I to III using the GEO database (No. GSE64591). As shown in Figure 1, the differential expression of miR‐34b‐3p was the most significant among all the miRNAs. We then examined the expression of miR‐34b‐3p in 512 lung adenocarcinoma samples (Table 1) and adjacent normal tissues by qRT‐PCR. As presented in Figure 2A, miR‐34b‐3p was significantly decreased in cancerous tissues compared with normal controls. Consistent with this result, the expression of miR‐34b‐3p was much lower in the two NSCLC cell lines (H1299 and A549) compared with the human bronchial epithelial cell line (BEAS‐2B) (Figure 2B). Taken together, these data suggest that miR‐34b‐3p is significantly down‐regulated in NSCLC tumour tissues and cell lines.

**Table 1:** Patients characteristics (n = 512)

Characteristics	Number (%)
Age (y)	
Median (range)	64.8 (48‐77)
Gender	
Male	304 (59.4)
Female	208 (40.6)
Clinical stage	
I	286 (55.9)
II	124 (24.2)
III	102 (19.9)
Histological sub‐classification	
Adenocarcinoma	512 (100)
Smoking status	
Non‐smoker (0 y)	104 (20.3)
Previous light smoker (1‐10 y)	60 (11.7)
Previous heavy smoker (>10 y)	143 (27.9)
Current smoker	205 (40.0)

### 3.2 MicroRNA‐34b‐3p suppresses NSCLC cell growth in vitro

**Figure 3:**
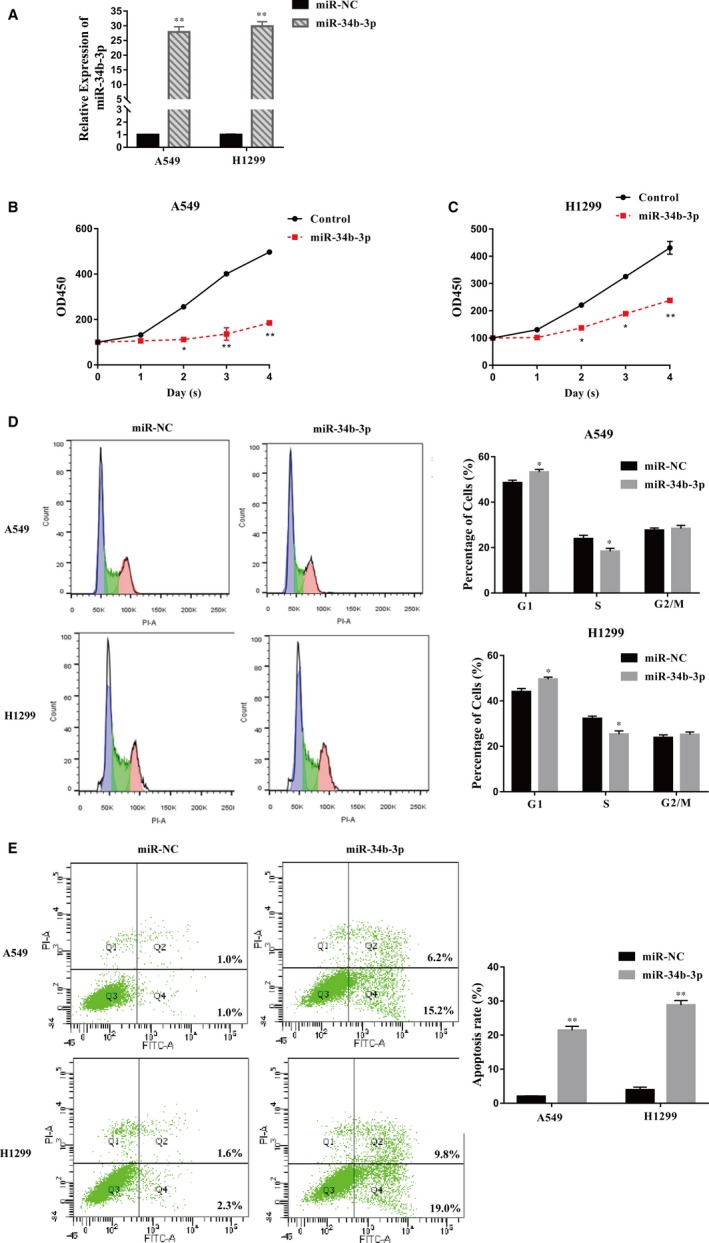
Overexpression of miR‐34b‐3p inhibited non‐small‐cell lung cancer cell growth. (A) miR‐34b‐3p expression levels in A549 or H1299 cells transfected with miR‐34b‐3p or miR‐NC were examined by qRT‐PCR. (B and C) Cell proliferation ability was compared between miR‐34b‐3p mimic‐ and NC‐transfected A549 and H1299 cells by the CCK‐8 assay. (D) Cell cycle analysis was performed in A549 and H1299 cells transfected with miR‐34b‐3p or miR‐NC. (E) Flow cytometry was performed in A549 and H1299 cells transfected with miR‐34b‐3p or miR‐NC to detect apoptosis. All experiments were performed in triplicate. *P < 0.05, **P < 0.01

We further investigated the biological function of miR‐34b‐3p in NSCLC cells. The expression of miR‐34b‐3p was obviously increased after transfection with the miR‐34b‐3p mimic compared with miR‐NC in H1299 and A549 cells (Figure 3A). The increased expression of miR‐34b‐3p resulted in a significant decrease in the viability of H1299 and A549 cells according to the CCK‐8 assay (Figure 3B,C). The up‐regulation of miR‐34b‐3p remarkably increased the number of cells in G1 phase and decreased the number of cells in S phase, leading to cell cycle arrest in H1299 and A549 cells compared with the control (Figure 3D). In addition, the overexpression of miR‐34b‐3p significantly promoted the apoptosis of H1299 and A549 cells (Figure 3E). These results indicate that miR‐34b‐3p negatively regulates the growth of NSCLC cells.

### 3.3 CDK4 is a direct target of miR‐34b‐3p

**Figure 4:**
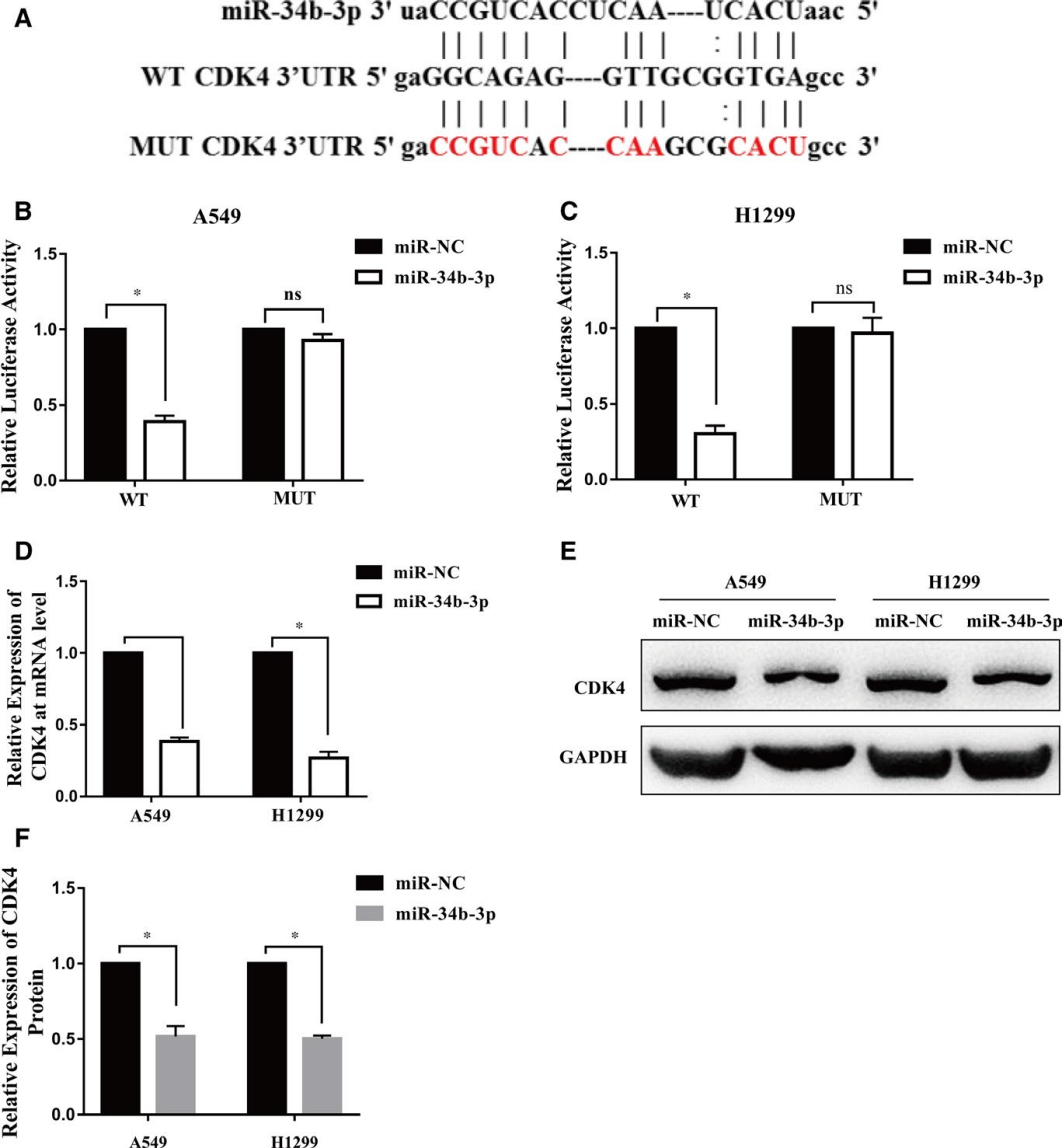
Cyclin‐dependent kinase 4 (CDK4) is a direct target of miR‐34b‐3p. (A) Potential interaction between miR‐34b‐3p and putative binding sites in the 3′‐UTR of CDK4 predicted by Starbase. (B and C) A luciferase reporter assay was performed in A549 and H1299 cells transfected with the miR‐34b‐3p mimic or miR‐NC together with luciferase reporter plasmids for 48 h. (D) The relative expression of CDK4 at the mRNA level in A549 and H1299 cells transfected with the miR‐34b‐3p mimic or miR‐NC was determined by qRT‐PCR. (E) The relative expression of CDK4 at the protein level in A549 and H1299 cells transfected with the miR‐34b‐3p mimic or miR‐NC was measured by Western blot. All experiments were performed in triplicate. (F) The protein level of CDK4 detected by Western blot was evaluated in grey scale. *P < 0.05

We used miRNA target prediction software to search for the target genes of miR‐34b‐3p. Interestingly, among the predicted targets, the complementary region of miR‐34b‐3p was identified in the 3′‐UTR of CDK4 (Figure 4A). Then, we constructed wild‐type CDK4 3′‐UTR and mutant CDK4 3′‐UTR luciferase reporter plasmids. MiR‐34b‐3p decreased luciferase activity compared to the control in both H1299 and A549 cells (Figure 4B,C). However, miR‐34b‐3p showed no effect on the mutant 3′‐UTR fragment (Figure 4B,C). Moreover, the mRNA and protein expression levels of CDK4 were significantly decreased in H1299 and A549 cells transfected with miR‐34b‐3p mimics (Figure 4D‐F). Taken together, these data indicate that CDK4 is a direct target of miR‐34b‐3p.

### 3.4 CDK4 is essential for NSCLC cell growth

**Figure 5:**
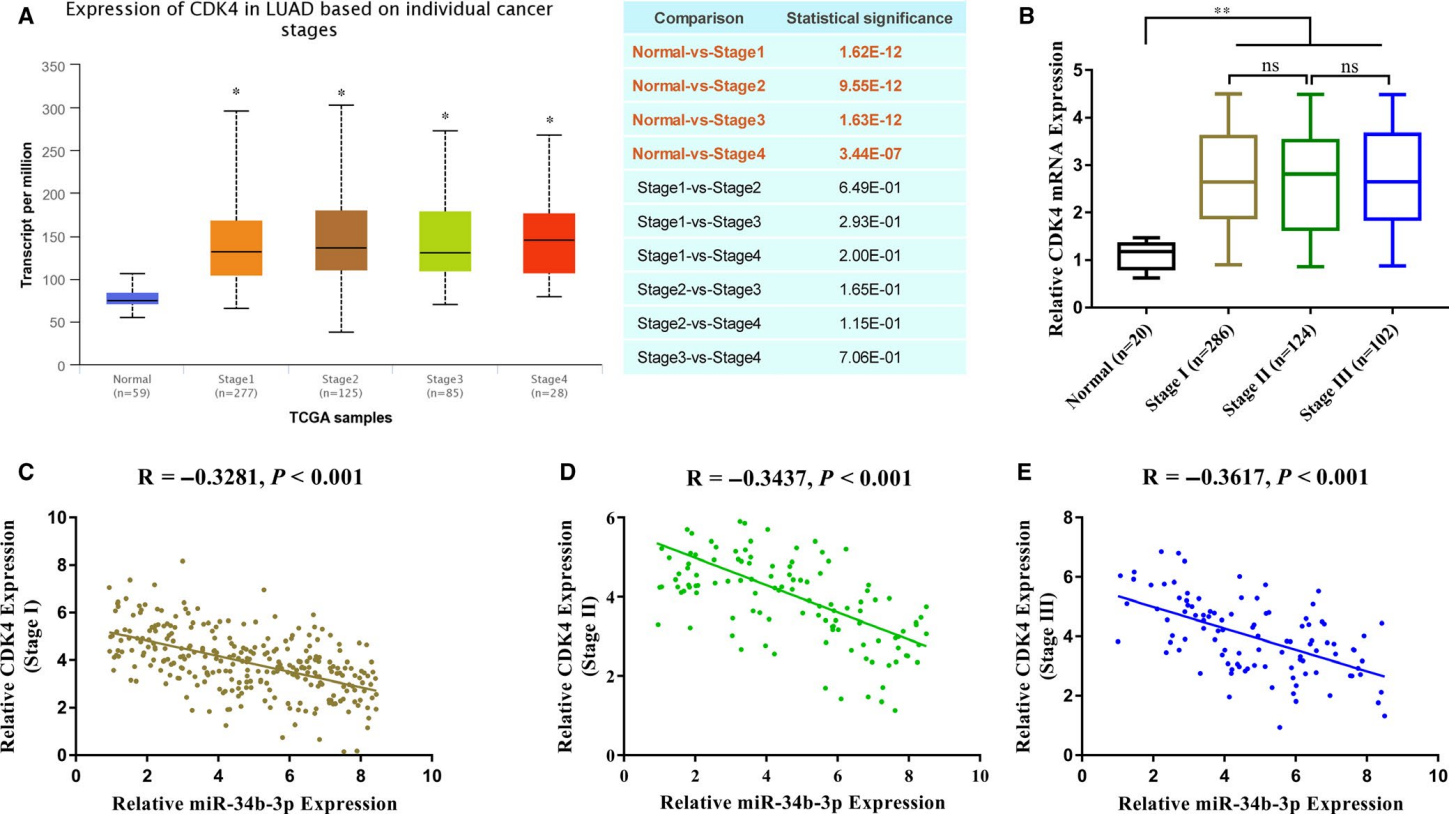
Expression of CDK4 mRNA in non‐small‐cell lung cancer (NSCLC) tissues. (A) CDK4 mRNA expression in lung adenocarcinoma tissues (different cancer stages) and normal tissues was analysed by the UALCAN (http://ualcan.path.uab.edu) online tool. (B) CDK4 mRNA expression in lung adenocarcinoma tissues (different cancer stages) and normal tissues was analysed by qRT‐PCR. (C, D, E) The expression of miR‐34b‐3p negatively correlated with CDK4 mRNA at different NSCLC disease stages. *P < 0.05, **P < 0.01

Next, we analysed the expression of CDK4 in NSCLC. First, we analysed the relevant data in The Cancer Genome Atlas (TCGA) library using the UALCAN (http://ualcan.path.uab.edu) online tool. CDK4 mRNA was highly expressed in lung cancer tissues as a whole but not significantly during different stages (Figure 5A). Subsequently, the relative content of CDK4 mRNA in 512 samples was detected by qPCR, and the results were similar to the database analysis (Figure 5B). There was a good negative correlation between the relative content of CDK4 mRNA and the relative expression of miR‐34b‐3p in different NSCLC stages (Figure 5C‐E).

**Figure 6:**
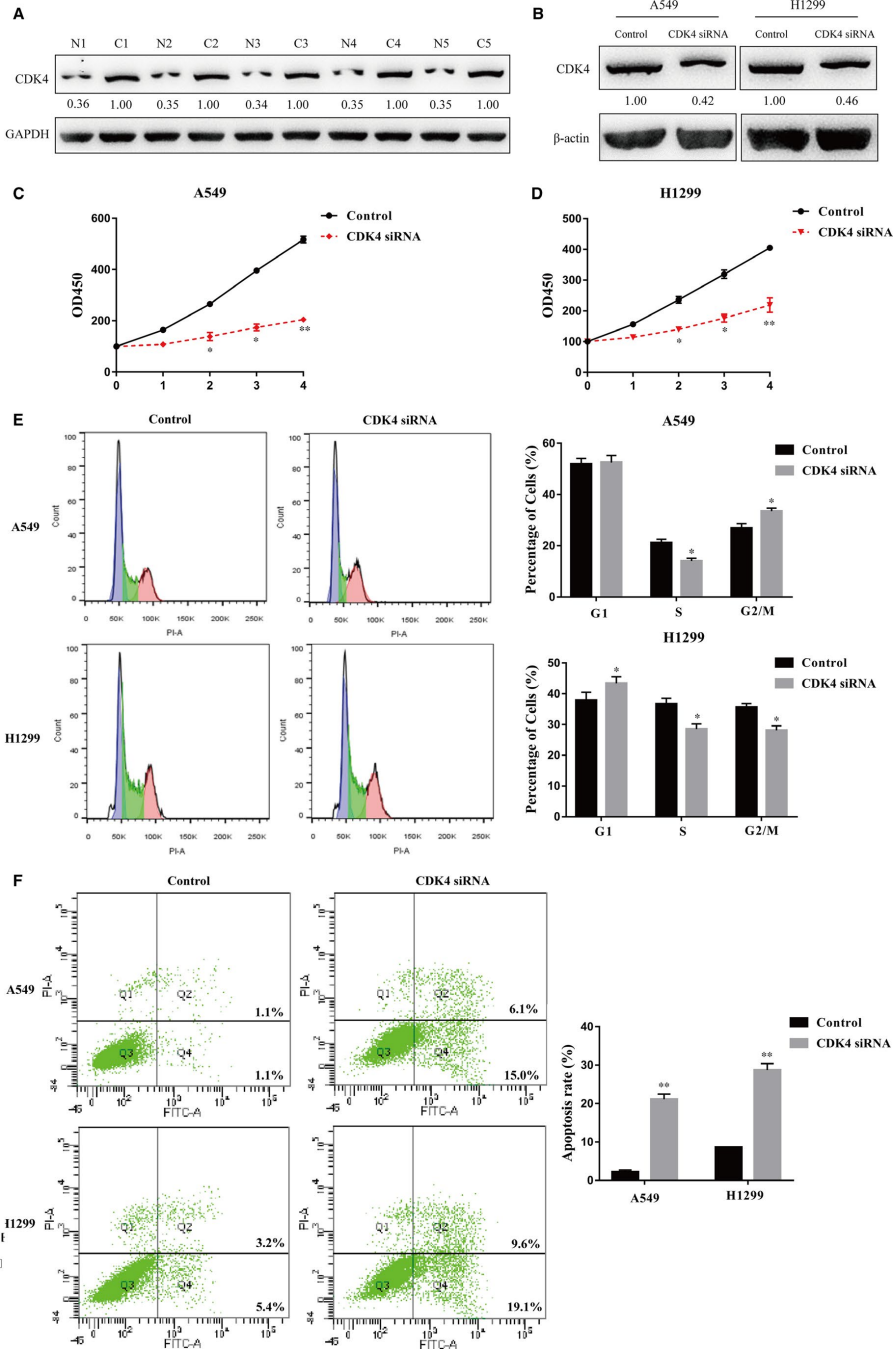
Effects of CDK4 knockdown on cell growth in non‐small‐cell lung cancer. (A) CDK4 expression in lung adenocarcinoma tissues (C) and adjacent normal tissues (N) was analysed by Western blot. (B) CDK4 expression in control siRNA‐ and CDK4 siRNA‐transfected A549/H1299 cells was measured by Western blot. (C) Cell proliferation was assessed in control siRNA‐ and CDK4 siRNA‐transfected A549/H1299 cells by the CCK‐8 assay. (D) Cell cycle distribution was examined in control siRNA‐ and CDK4 siRNA‐transfected A549/H1299 cells. (E) Flow cytometry was performed in control siRNA‐ and CDK4 siRNA‐transfected A549/H1299 cells to detect apoptosis. All experiments were performed in triplicate. *P < 0.05, **P < 0.01

We next measured the expression levels of CDK4 in human NSCLC samples by Western blot analysis. CDK4 was remarkably up‐regulated in NSCLC tissues compared to adjacent normal tissues (Figure 6A). We utilized a siRNA to knockdown CDK4 in NSCLC cell lines, and the results showed a high knockdown efficiency of CDK4 in H1299 and A549 cells at the protein level (Figure 6B). The CCK‐8 assay showed that the viability of H1299 and A549 cells transfected with the siRNA targeting CDK4 was significantly decreased (Figure 6C,D), which was identical to the phenotypes that resulted from miR‐34b‐3p overexpression (Figure 3B,C). In addition, cell cycle analysis showed that the reduced expression of CDK4 increased the percentages of G1 cells and decreased the subpopulation of S cells, leading to cell cycle arrest at S phase (Figure 6E). Cell apoptosis detection showed that more cells underwent apoptosis with CDK4 knockdown (Figure 6F). Our data reveal that CDK4 might function as an oncogene in NSCLC by promoting cell proliferation, shifting cell cycle distribution from G1 to S phase and repressing cell apoptosis.

### 3.5 MicroRNA‐34b‐3p represses lung cancer cell growth by targeting CDK4

**Figure 7:**
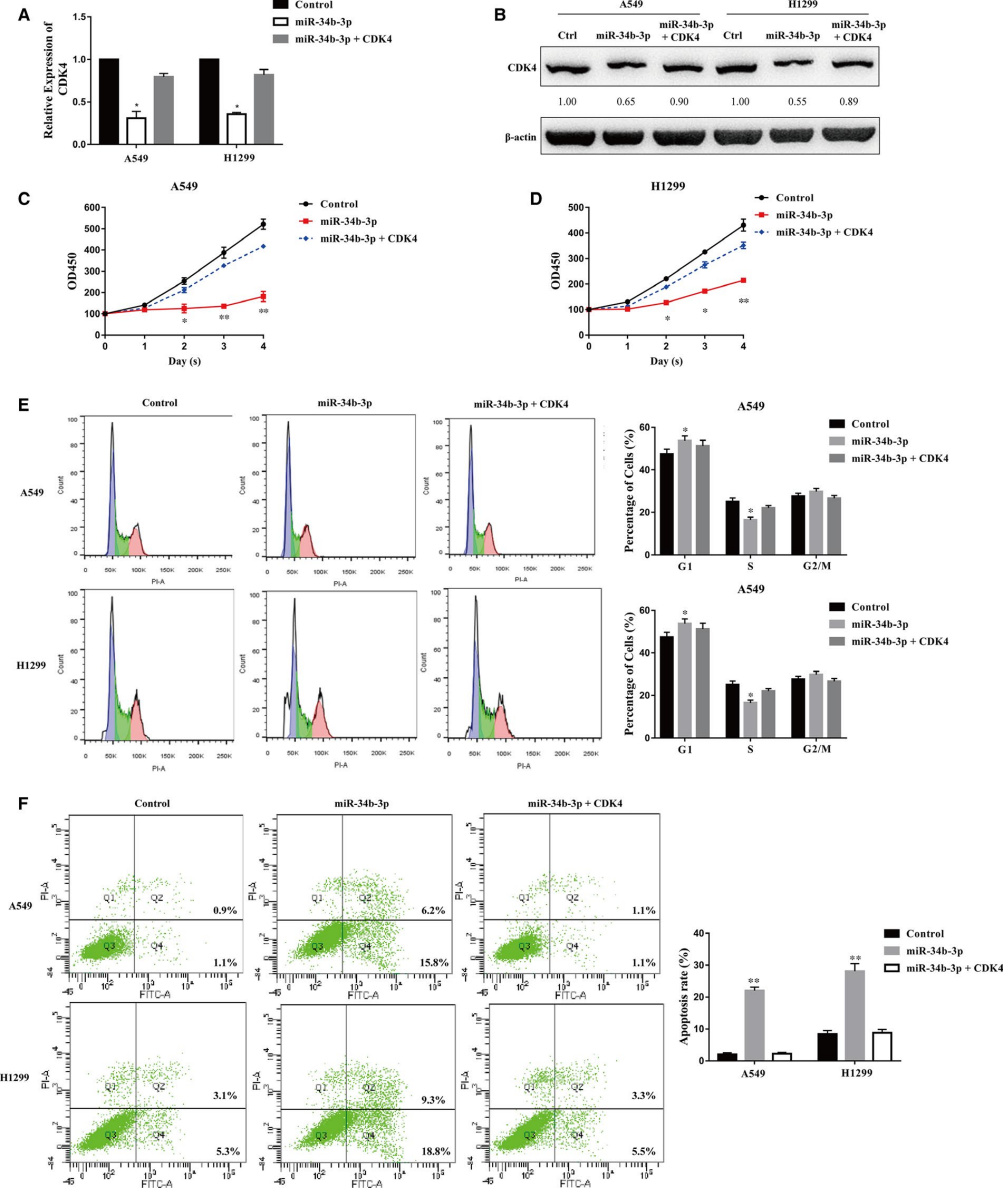
miR‐34b‐3p repressed lung cancer cell growth by targeting CDK4. (A) The relative expression of CDK4 at the mRNA level was measured in control miRNA‐overexpressing or miR‐34b‐3p‐overexpressing A549/H1299 cells with/without CDK4 overexpression by qRT‐PCR. (B) The expression of CDK4 at the protein level was measured in control miRNA‐overexpressing or miR‐34b‐3p‐overexpressing A549/H1299 cells with/without CDK4 overexpression by Western blot. (C and D) Cell proliferation was assessed in control miRNA‐overexpressing or miR‐34b‐3p‐overexpressing A549/H1299 cells with/without CDK4 overexpression by the CCK‐8 assay. (E) Cell cycle distribution was examined in control miRNA‐overexpressing or miR‐34b‐3p‐overexpressing A549/H1299 cells with/without CDK4 overexpression. (F) Flow cytometry was performed in control miRNA‐overexpressing or miR‐34b‐3p‐overexpressing A549/H1299 cells with/without CDK4 overexpression to detect apoptosis. All experiments were performed in triplicate. *P < 0.05, **P < 0.01

To confirm our hypothesis that miR‐34b‐3p functions by directly targeting CDK4, we co‐overexpressed CDK4 together with miR‐34b‐3p in H1299 and A549 cells. miR‐34b‐3p overexpression impaired the expression of CDK4, while the co‐overexpression of miR‐34b‐3p and CDK4 restored CDK4 expression at the mRNA and protein levels in both H1299 and A549 cells (Figure 7A,B). The co‐overexpression of miR‐34b‐3p and CDK4 restored the cell growth of NSCLC, indicating that miR‐34b‐3p functions directly through CDK4 (Figure 7C,D). We also performed cell cycle and flow cytometry analyses of cells ectopically expressing miR‐34b‐3p and CDK4. The increase in the G1 subpopulation and the decrease in the S subpopulation resulting from ectopic miR‐34b‐3p expression were attenuated by CDK4 overexpression (Figure 7E). Moreover, the increase in apoptotic cells induced by miR‐34b‐3p was reversed by cotransfection with miR‐34b‐3p and CDK4 (Figure 7F). In conclusion, these results suggest that miR‐34b‐3p suppresses NSCLC cell growth by directly targeting the expression of CDK4.

## 4 DISCUSSION

Lung cancer is the most common cancer worldwide and has a poor prognosis and high possibility of tumour metastasis. Despite the tumourigenesis and pathophysiology of NSCLC, great achievements have been made during the past few years.^8^, ^9^ The underlying mechanism of NSCLC carcinogenesis remains elusive. Thus, a comprehensive and profound understanding of these mechanisms will promote the development of novel therapeutic targets for NSCLC treatment. In the present study, we focused on miR‐34b‐3p, which was decreased in a series of cancers. We found that miR‐34b‐3p was also down‐regulated in lung adenocarcinoma tissues and cell lines. In addition, the overexpression of miR‐34b‐3p suppressed cell proliferation, blocked the cell cycle in G1 phase and promoted cell apoptosis, indicating that miR‐34b‐3p negatively regulates NSCLC cell growth.

The microRNA (miR)‐34 family is composed of 5p and 3p strands of miR‐34a, miR‐34b and miR‐34c.^10^ *Córdova‐Rivas S et al* found that the 5p and 3p strands of miR‐34 family members had differential effects on cell proliferation, migration and invasion in cervical cancer cells. In our research, we focused on the biological effects of miR‐34b‐3p on lung adenocarcinoma proliferation, cell cycle progression and cell apoptosis. The effects of miR‐34b‐3p on lung adenocarcinoma cell migration and invasion were examined as part of our ongoing research in another project. The function of the 5p strand of miR‐34b and other members of the miR‐34 family in lung adenocarcinoma should be explored in future studies.

Aberrant miRNA expression is related to various biological processes, such as proliferation, apoptosis, angiogenesis, migration and invasion. Human miR‐34b‐3p is down‐regulated in several cancer types, such as small lung cancer cell (SCLC), breast cancer and prostate cancer.^11^, ^12^, ^13^, ^14^ This observation suggests that miR‐34b‐3p might play a key role in tumourigenesis. Recent studies have shown an association between increased miR‐34b‐3p expression and early fibrosis in HBV‐infected liver disease.^15^ miR‐34b‐3p is down‐regulated in small‐cell lung cancer and is a candidate antitumour miRNA.^11^ MicroRNA‐34b potently inhibited migration and invasion in metastatic prostate cancer cells by regulating the TGF‐β pathway.^12^ MicroRNA‐34b acted as a tumour suppressor in the oestrogen‐dependent growth of breast cancer cells.^13^ These findings prompted us to investigate the function and regulatory mechanism of miR‐34b‐3p in NSCLC cells. In this study, miR‐34b‐3p negatively regulated cell growth in NSCLC. Cyclin‐dependent kinase 4 (CDK4) was confirmed to be directly targeted by miR‐34b‐3p in NSCLC cells. Moreover, the overexpression of CDK4 reversed the cell growth suppression blocked by ectopic miR‐34b‐3p expression in vitro.

Cyclin‐dependent kinase 4 (CDK4) was reported to be important for cell cycle G1 phase progression.^16^ The activity of the kinase complex was restricted to G1‐S phase, which is controlled by the regulatory subunits of D‐type cyclins and the cyclin‐dependent kinase (CDK) inhibitor p16 (INK4a).^17^, ^18^ The deregulation of CDK4 expression is associated with several aspects of tumourigenesis, including cell cycle arrest, proliferation and abnormal apoptosis.^18^, ^19^ The down‐regulation of cyclin D1 and CDK4 suppressed human colorectal cancer cell proliferation.^20^ In this study, CDK4 was overexpressed in human lung adenocarcinoma samples. Cell proliferation inhibition is mediated by cell cycle arrest and apoptosis with CDK4 knockdown. The overexpression of miR‐34b‐3p inhibited NSCLC cell growth, which was restored by CDK4 overexpression. Our discovery that miR‐34b‐3p inhibits CDK4 expression and is down‐regulated in NSCLC might shed light on the prognosis and treatment of NSCLC.

In conclusion, we identified that miR‐34b‐3p negatively regulates NSCLC cell growth by targeting CDK4. The overexpression of miR‐34b‐3p may have potential therapeutic applications in NSCLC. The application of miRNAs to the treatment of NSCLC will be investigated in future studies.

## CONFLICT OF INTEREST

The authors declare no potential conflicts of interest with respect to the research, authorship and/or publication of this article.

## DATA AVAILABILITY STATEMENT

The data used to support the findings of this study are available from the corresponding author upon request.
